# Genomic characterization of antimicrobial resistance, virulence, and transmission of non-typhoidal *Salmonella* from retail red meat in Hunan, China

**DOI:** 10.3389/fmicb.2026.1840000

**Published:** 2026-05-29

**Authors:** Shuai Chen, Yating Ma, Wansi Zhang, Tianbing Lai, Linqing Zhang, Qing Yuan, Zhifei Zhan, Fang Liu

**Affiliations:** Hunan Provincial Center for Disease Control and Prevention (Hunan Academy of Preventive Medicine), Changsha, China

**Keywords:** antimicrobial resistance, cgMLST, China, retail red meat, *Salmonella*, typhoid toxin, virulence factors, whole-genome sequencing

## Abstract

**Introduction:**

*Salmonella* is a major foodborne pathogen frequently transmitted via retail meat. This study comprehensively characterized the genomic features of non-typhoidal *Salmonella* from retail red meat in Hunan, China, including antimicrobial resistance, virulence determinants, and transmission patterns.

**Methods:**

We characterized 147 *Salmonella* isolates from retail red meat (pork, beef, and mutton) collected in Hunan, China (2021 and 2023) using whole-genome sequencing and antimicrobial susceptibility testing to investigate their resistance and genomic characteristics.

**Results:**

High resistance rates were observed: 115 isolates (78.2%) were resistant to at least one agent, and 92 (62.6%) were multidrug-resistant (MDR). The predominant serotypes were *Salmonella* London (25.2%) and *S*. Typhimurium (24.5%). Critically, the 16S rRNA methyltransferase gene *rmtB* was identified in three isolates (2.0%)—the only amikacin-resistant isolates. The extended-spectrum β-lactamase (ESBL) gene *bla*_CTX-M-55_ was detected in six isolates (4.1%), and plasmid-mediated AmpC genes (*bla*_CMY-2_/*bla*_DHA-1_) in three (2.0%). Of major concern, the complete typhoid toxin gene cluster (*cdtB, pltA, pltB*) was identified in 14 non-typhoidal *Salmonella* (NTS) isolates (9.5%) across diverse serotypes. Core-genome multilocus sequence typing (cgMLST) revealed 22 genomic clusters, demonstrating multi-level transmission from regional sources to local outbreaks.

**Discussion:**

These findings highlight the emergence of multidrug-resistant *Salmonella* clones carrying clinically significant resistance and virulence determinants in retail red meat, underscoring the need for enhanced food chain surveillance.

## 1 Introduction

*Salmonella* is one of the leading causes of foodborne bacterial diseases worldwide, with retail meat products serving as major transmission vehicles to humans (Bolkenov et al., 2024). The emergence and dissemination of antimicrobial-resistant *Salmonella*, particularly multidrug-resistant (MDR) strains, pose significant threats to public health and clinical treatment (Habib et al., 2024; Li et al., 2022).

In China, the world's largest producer and consumer of meat (ReportLinker, 2024), *Salmonella* contamination and antimicrobial resistance in retail meat are of great concern. Genomic surveillance has revealed diverse antimicrobial resistance and virulence profiles in *Salmonella* from various sources across the country (Guo et al., 2023). However, comprehensive genomic data on *Salmonella* from retail meat in Hunan Province—a major agricultural and food-producing region in central China (Hunan Provincial Bureau of Statistics, 2024)—remain limited. The prevalence, antimicrobial resistance profiles, and virulence characteristics of *Salmonella* in this region have not been systematically investigated, hindering a full understanding of local transmission dynamics, resistance evolution, and potential pathogenic risks.

Given the limited comprehensive genomic data available for *Salmonella* from retail red meat in Hunan Province, this study aimed to systematically characterize non-typhoidal *Salmonella* isolates using whole-genome sequencing. We analyzed the population structure, antimicrobial resistance phenotypes and their genetic determinants, virulence gene repertoires, and transmission patterns of high-risk clones by integrating cgMLST analysis with epidemiological data. This study provides critical genomic insights into the epidemiological characteristics, resistance evolution, and virulence potential of *Salmonella* in retail red meat in Hunan, supporting evidence-based food safety risk assessment and management.

## 2 Materials and methods

### 2.1 Strain source and collection

**Figure 1:**
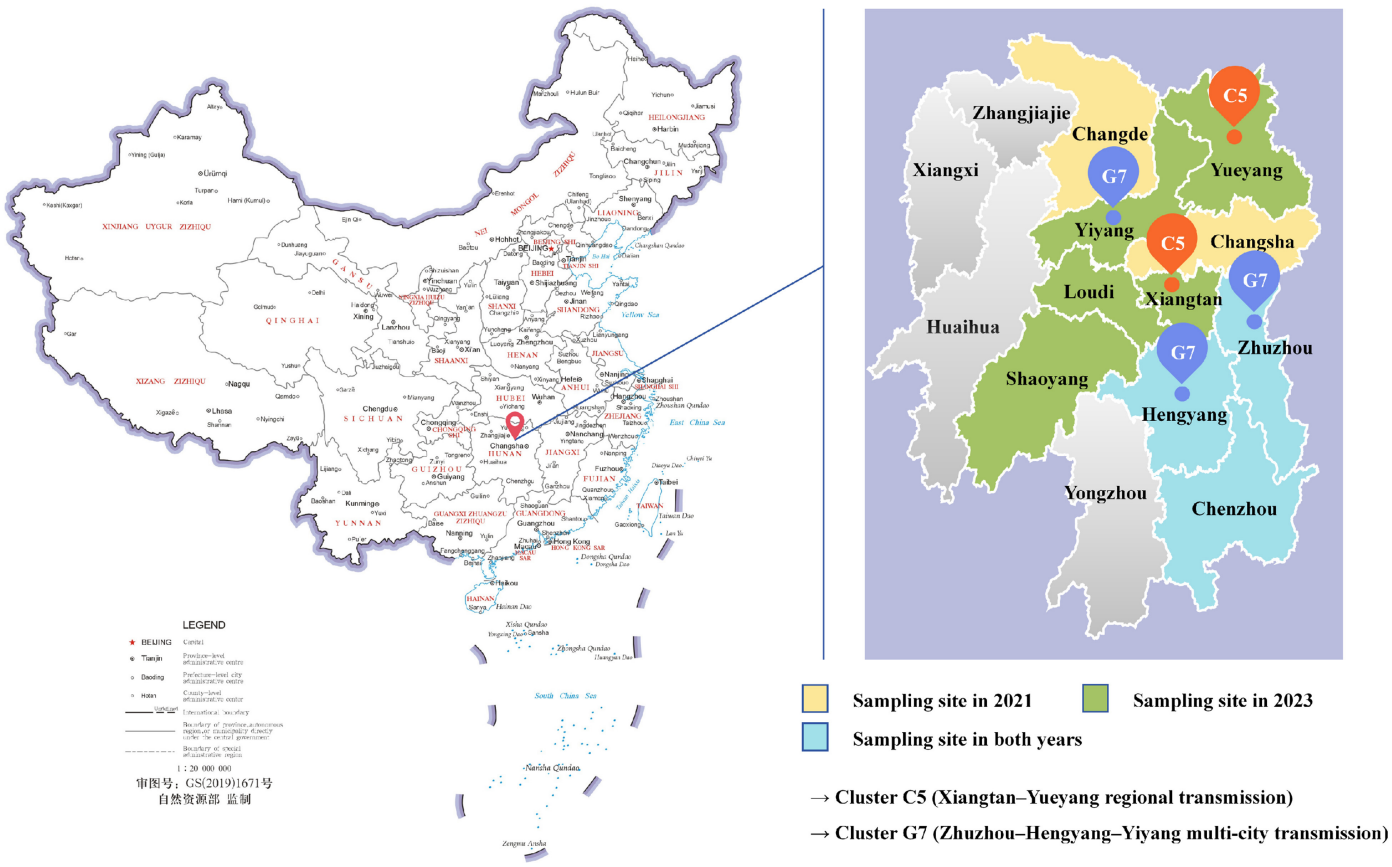
Geographic distribution of sampling sites and major transmission clusters of *Salmonella* in Hunan Province, China.

As part of the national food safety risk surveillance program, a total of 1,529 retail red meat samples were collected from farmers' markets, supermarkets, and retail stores across 10 prefecture-level cities in Hunan Province, China, in 2021 and 2023 (red meat was not sampled in 2022 due to an alternating surveillance design targeting other food types that year). The samples included fresh, chilled, frozen, raw seasoned, and cooked products (excluding commercially sterile products) derived from pork, beef, and mutton. From these samples, 147 *Salmonella* isolates were recovered and subjected to further analysis. Due to the project's task allocation, sampling did not cover all 14 prefectures of Hunan Province. The coverage varied by year, with some prefectures participating in both years and others in only one year (Figure 1).

Detailed information on all 147 isolates, including serotypes, STs, sampling locations, and dates, is provided in Supplementary Table S1.

### 2.2 *Salmonella* isolation and identification

*Salmonella* isolation was performed by municipal Centers for Disease Control and Prevention according to the national standard GB 4789.4-2016 (Standardization Administration of China, 2016). Briefly, samples were pre-enriched in buffered peptone water, followed by selective enrichment in tetrathionate broth (TTB) and selenite cystine broth (SC), and then streaked onto bismuth sulfite (BS) agar and xylose lysine deoxycholate (XLD) agar. Presumptive colonies were confirmed using biochemical tests and serological agglutination.

All isolates were then transferred to the provincial CDC for confirmation and further analysis. Upon receipt, each isolate was re-confirmed using the VITEK 2 (bioMérieux, France) automated microbial identification system. Serotyping was performed by slide agglutination using commercial antisera (Statens Serum Institut (SSI), Copenhagen, Denmark; Ningbo Tianrun Biological Pharmaceutical Company Ltd., Ningbo, China) following the White–Kauffmann–Le Minor scheme (Grimont and Weill, 2007). Subsequently, all confirmed isolates were subjected to antimicrobial susceptibility testing and whole-genome sequencing at the provincial CDC.

### 2.3 Antimicrobial susceptibility testing

Antimicrobial susceptibility was determined by the broth microdilution method using the AutoMic-i600 (Autobio, China) automatic susceptibility testing system according to the manufacturer's instructions, using the corresponding gram-negative susceptibility panels (Autobio, China). A panel of 24 antimicrobial agents from 10 classes was selected based on recommendations from national and international surveillance networks (e.g., NARMS, CARSS, CHINET, Pathogen Identification Network) and guidelines from the Clinical and Laboratory Standards Institute (CLSI), the U.S. Food and Drug Administration (FDA), and the European Committee on Antimicrobial Susceptibility Testing (EUCAST).

The tested antimicrobials included: penicillins (ampicillin); β-lactam/β-lactamase inhibitor combinations (ampicillin/sulbactam, ceftazidime/avibactam); cephalosporins (cefazolin, cefuroxime, cefotaxime, ceftazidime, cefepime, ceftiofur); cephamycins (cefoxitin); carbapenems (imipenem, meropenem, ertapenem); aminoglycosides (gentamicin, streptomycin, amikacin); polymyxins (colistin, polymyxin B); tetracyclines (tetracycline, tigecycline); fluoroquinolones (ciprofloxacin); phenicols (chloramphenicol, florfenicol); and folate pathway inhibitors (trimethoprim/sulfamethoxazole).

The minimal inhibitory concentration (MIC) for each antimicrobial was determined and interpreted using a unified set of breakpoints compiled from the following publicly available standards: CLSI M100-S34 (Clinical and Laboratory Standards Institute, 2024), CLSI M45-A3 (Clinical and Laboratory Standards Institute, 2016), CLSI VET01-A4 (Clinical and Laboratory Standards Institute, 2013), FDA (U.S. Food and Drug Administration, 2024), and EUCAST (European Committee on Antimicrobial Susceptibility Testing, 2024). To ensure transparency and reproducibility, the complete breakpoint table is provided in Supplementary Table S2. All isolates were interpreted consistently using this unified reference, regardless of collection year. *Escherichia coli* ATCC 25922 was used as the quality control strain. Isolates resistant to three or more antimicrobial classes were defined as MDR (Magiorakos et al., 2012).

### 2.4 Whole-genome sequencing and data processing

Genomic DNA was extracted using the TIANamp Bacteria DNA Kit (TIANGEN, China) according to the manufacturer's instructions. Whole-genome sequencing was performed at Novogene Bioinformatics Technology Company Ltd. (Beijing, China) using the Illumina NovaSeq 6000 platform (Illumina, USA) with paired-end 150 bp (PE150) chemistry. Raw reads were processed to obtain clean data by removing adapter sequences and low-quality reads using Fastp (Chen et al., 2018). The clean reads were assembled using SPAdes v3.15.0 (Bankevich et al., 2012) with default parameters. Sequencing quality was assessed by evaluating sequencing depth, genome coverage, Q20/Q30 values, and assembly statistics (contig counts and N50). Assemblies meeting quality thresholds for contig count and single-base error rate were used for downstream analyses. Detailed quality metrics for each isolate are provided in Supplementary Table S3.

### 2.5 Bioinformatic analysis

Clean reads were uploaded to the Microobench platform (Beijing Zhongwei Shuchuang Technology Company Ltd., China), a pathogen microbial analysis workstation, for downstream bioinformatic analyses. *In silico* serotyping was performed using SeqSero2 (https://github.com/denglab/SeqSero2). Multilocus sequence typing (MLST) was performed based on seven housekeeping genes (*aroC, dnaN, hemD, hisD, purE, sucA, thrA*) using the PubMedST database (Jolley and Maiden, 2010). Virulence genes were identified by alignment against the Virulence Factor Database (VFDB) (Chen et al., 2016; Liu et al., 2019) with a minimum threshold of 97% sequence identity and 97% coverage. Antimicrobial resistance genes were identified by alignment against the Comprehensive Antibiotic Resistance Database (CARD) (Alcock et al., 2023) using the same thresholds. These thresholds were the default parameters of the Microobench platform.

CgMLST analysis was performed to investigate the genetic relatedness among isolates. A clustering threshold of ≤ 10 allele differences was applied to define genomic clusters, consistent with previous studies on *Salmonella enterica* outbreak investigations (Pearce et al., 2018; Trees et al., 2024). Based on the available sampling information (collection date, city, county, market, and sample type), the identified clusters were further categorized into three descriptive types: (E) potential epidemiologically linked clusters (isolates with clear spatiotemporal proximity, e.g., same market or same-day sampling from the same city); (C) common source-supported clusters (isolates falling within the same genomic cluster but lacking strong spatiotemporal links, suggesting potential shared upstream sources); and (G) genomic clusters only (isolates that clustered genomically but had no identifiable spatiotemporal or source links, representing background lineages). This classification is descriptive and does not imply confirmed transmission events, as cross-contamination during sampling or laboratory processing cannot be completely excluded.

## 3 Results

### 3.1 Distribution of *Salmonella* isolates

The 147 *Salmonella* isolates were categorized into five serogroups (B, C, D, E, and F), with serogroup B being the most prevalent (41.5%, 61/147), and further differentiated into 24 distinct serotypes (Table 1). The population exhibited a highly skewed distribution, with two serotypes dominating the collection: *S*. London (25.2%, 37/147) and *S*. Typhimurium (24.5%, 36/147; including both the typical serotype and its monophasic variant *S*. 1, 4, [5], 12: i:–. These were followed by *S*. Rissen (14.3%) and *S*. Derby (12.2%).

**Table 1:** Characteristics of the dominant *Salmonella* clones isolated from retail meat.

Serotype	Serogroup	Predominant ST(s)	No. (%)	Main source
*S*. London	E	ST155 (36), ST14132^e^ (1)	37 (25.2)	RM^a^, CM^b^
*S*. Typhimurium^c^	B	ST34 (26), ST19 (8), ST14131^e^ (2)	36 (24.5)	RM, CM
*S*. Rissen	C	ST469 (21)	21 (14.3)	RM
*S*. Derby	B	ST40 (16), ST71 (2)	18 (12.2)	RM
*S*. Give	E	ST516 (4), ST654 (3)	7 (4.8)	RM, CM
*S*. Goldcoast	C	ST358 (4)	4 (2.7)	RM, CM
Others^d^	–	Various	24 (16.3)	–
Total	–	27 STs	147 (100)	–

^*a*^Raw meat (including fresh, chilled, frozen, and seasoned raw meat products).

^*b*^Cooked meat products. Sources are listed in order of descending frequency.

^*c*^*S*. Typhimurium includes both the typical serotype and its monophasic variant *S*. 1, 4, [5], 12: i:–.

^*d*^The “Others” category includes 18 serotypes: *S*. Agona, *S*. Altona, *S*. Anatum, *S*. Braenderup, *S*. Corvallis, *S*. Hadar, *S*. Indiana, *S*. Kentucky, *S*. Liverpool, *S*. Livingstone, *S*. Mbandaka, *S*. Meleagridis, *S*. Newport, *S*. Panama, *S*. Rubislaw, *S*. Schwarzengrund, *S*. Senftenberg, and *S*. Stanley. These isolates represented 16 distinct STs (detailed in Supplementary Table S1).

^*e*^ST14131 and ST14132 are novel sequence types assigned via EnteroBase.

The WGS-predicted serotypes obtained by SeqSero2 were compared with the traditional slide agglutination results (Supplementary Table S1). SeqSero2 failed to predict any serotype for three isolates (2.0%). For four isolates (2.7%), the prediction was ambiguous (“Goldcoast or Brikama”). Among the remaining 140 isolates (95.2%) with a definitive WGS prediction, 132 (94.3%) were concordant with the traditional serotype, and eight (5.7%) were discordant. All discordant cases involved *S*. Typhimurium and its monophasic variant *S*. 1, 4, [5], 12: i:–, where SeqSero2 and slide agglutination gave opposite assignments.

Multilocus sequence typing (MLST) analysis identified 29 sequence types (STs) among the isolates, including two novel STs: ST14131 and ST14132. Notably, each predominant serotype was closely associated with one to three key STs (Table 1). For instance, *S*. London isolates almost exclusively belonged to ST155 (36/37), with one additional isolate representing the novel ST14132; *S*. Typhimurium was predominantly ST34 (26/36) and ST19 (8/36), with two additional isolates representing the novel ST14131.

### 3.2 Virulence gene profiles and key determinants

The virulence gene repertoire (virulome) of all 147 isolates was systematically analyzed according to the VFDB classification (Table 2).

**Table 2:** Prevalence of virulence factors based on VFDB classification.

VFDB category	Virulence factor/system	No. (%)	Key function
Adherence	Core adhesins (six genes: *agf, bcf, sinH, stb, sth*, type 1 fimbriae)	147 (100)	Essential for biofilm formation and host colonization
	High-frequency adhesins (*std, sti, stf, misL*)	134–145 (91.2–98.6)	Enhanced intestinal colonization
	Mid-frequency adhesins (*saf, ratB, lpf, siiE, ompD, stj*)	77–121 (52.4–82.3)	Niche-specific colonization
	Low-frequency adhesins (*stc, peg, ste, tcf, shdA, sta, stk*, P fimbriae)	2–72 (1.4–49.0)	Accessory roles in specific contexts
	Intestinal persistence factor (*shdA*)	43 (29.3)	Co-localized with all 37 *S*. London
Effector delivery system	SPI-1 and SPI-2 TTSS apparatus	147 (100)	Structural components of type III secretion systems
	System-specific TTSS effectors (TTSS-1, TTSS-2)	147 (100)	Epithelial cell invasion and intracellular survival
	TTSS effectors secreted via both systems	117 (79.6)	Enhanced flexibility in host manipulation
	SCI-encoded T6SS and its effectors	140 (95.2)	Bacterial competition and host interaction
	*apeE*	146 (99.3)	Outer membrane esterase
	T6SS (non-SCI encoded)	4 (2.7)	Rare accessory system
Invasion	*pagN, tia*	147 (100), 2 (1.4)	Host cell invasion
Motility	Peritrichous flagella	147 (100)	Motility and chemotaxis
Nutritional/metabolic	*mgtBC*, salmochelin, iron/manganese transport	147 (100), 147 (100), 1 (0.7)	Survival under nutrient-limited conditions
Regulation	*fur, phoPQ, rcsAB, rpoS*	147 (100)	Coordination of virulence gene expression
Stress survival	*sodCI*	63 (42.9)	Resistance to oxidative stress
Antimicrobial/competitive	*mig-14*	144 (98.0)	Resistance to antimicrobial peptides
Exotoxin	Typhoid toxin (complete: *cdtB/pltA/pltB*)	14 (9.5)	Complete cluster in multiple serotypes
	Typhoid toxin (partial: *cdtB/pltA*)	1 (0.7)	Defective cluster (lacking *pltB*); *S*. Panama ST48
	*clyA*	15 (10.2)	Cytolytic toxin
	Colicin Ib	3 (2.0)	Bacteriocin for bacterial competition

TTSS, type III secretion system; T6SS, type VI secretion system; SPI, *Salmonella* pathogenicity island; SCI, *Salmonella* centisome island; NTS, non-typhoidal *Salmonella*.

#### 3.2.1 Adherence genes

Adherence genes constituted the most abundant category. A core set of six adhesins (*agf, bcf, sinH, stb, sth*, and type 1 fimbriae) was present in all isolates. Multiple additional adhesins were detected at frequencies ranging from 98.6 to 1.4%. Critically, the intestinal persistence factor *shdA* was identified in 43 isolates (29.3%), including all 37 *S*. London isolates.

#### 3.2.2 Effector delivery systems

Effector delivery systems were highly prevalent. The type III secretion system (TTSS) apparatus encoded on SPI-1 and SPI-2, along with their system-specific secreted effectors, were present in all isolates. TTSS effectors capable of secretion via both systems were found in 117 isolates (79.6%). The SCI-encoded type VI secretion system (T6SS) and its effectors were identified in 140 isolates (95.2%). The outer membrane esterase *apeE* was detected in 146 isolates (99.3%), while a distinct non-SCI T6SS was present in only four isolates (2.7%).

#### 3.2.3 Nutritional/metabolic and stress survival systems

Nutritional/metabolic and stress survival systems were complete. All isolates carried the magnesium transport system *mgtBC*, the siderophore salmochelin, and global regulators including *fur, phoP/Q, rcsAB* and *rpoS*. Macrophage survival factors *mig-14* and *sodCI* were detected in 144 (98.0%) and 63 (42.9%) isolates, respectively.

#### 3.2.4 Invasion and motility factors

The invasion-associated gene *pagN* was present in all isolates (147, 100%), while *tia* was detected in two isolates (1.4%). All isolates carried genes for peritrichous flagella.

#### 3.2.5 Typhoid toxin gene cluster

Of major concern, the complete typhoid toxin gene cluster (*cdtB, pltA, pltB*) was identified in 14 NTS isolates (9.5%) across diverse serotypes, including *S*. Give (ST516/ST654), *S*. Goldcoast (ST358), *S*. Rubislaw (ST820), *S*. Schwarzengrund (ST96), and *S*. Indiana (ST17). A single *S*. Panama (ST48) isolate carried a defective unit (*cdtB* and *pltA* only).

### 3.3 Antimicrobial resistance phenotypes and their genetic determinants

Antimicrobial susceptibility testing revealed high rates of resistance among the 147 *Salmonella* isolates, with 115 isolates (78.2%) resistant to at least one agent. Of major concern, 92 isolates (62.6%) were MDR (Table 3).

**Table 3:** Antimicrobial resistance phenotypes and genetic determinants by antibiotic usage category.

Usage category	Drug class	Antimicrobial agent	*R*/*I* isolates (%)^a^	Key determinant(s) (%)^a^
Veterinary only	Phenicol	Florfenicol	74 *R* (50.3)	*floR* (94.6)
	Cephalosporin	Ceftiofur	8 *R* (5.4), 2 *I* (1.4)^b^	*bla*_CTX-M-55_ (60.0), *bla*_TEM-1_ (50.0), *bla*_LAP-2_ (30.0), *bla*_CMY-2_ (20.0), *bla*_DHA-1_ (10.0)
Human and veterinary shared	Tetracycline	Tetracycline	99 *R* (67.3)	*tet*(*A)* (4.0), *tet(B)* (23.2), *tet(D)* (1.0), *tetR* (23.2)
	Penicillin	Ampicillin	87 *R* (59.2)	*bla*_TEM-1_ (83.9), *bla*_CTX-M-55_ (6.9), *bla*_LAP-2_ (3.4), *bla*_CMY-2_ (2.3), *bla*_DHA-1_ (1.1)
	Aminoglycoside	Streptomycin	94 *R* (63.9)	*aph(6)-Id* (47.9), *aadA* (33.0), *aadA2* (28.7), *aph(3″)-Ib* (27.7), *aadA16* (20.2)
	Aminoglycoside	Gentamicin	32 *R* (21.8)	*aac(3)-IId* (78.1), *aph(6)-Id* (78.1), *aph(3″)-Ib* (62.5)
	Folate inhibitor	Trimethoprim-sulfamethoxazole	68 *R* (46.3)	*sul2* (70.6), *sul3* (58.8), *sul1* (36.8), *dfrA12* (50.0), and others^c^
	Phenicol	Chloramphenicol	77 *R* (52.4)	*floR* (89.6), *cmlA1* (39.0), and others^d^
	β-lactam/BI	Ampicillin/sulbactam	61 *R* (41.5)	–
Human only	Cephalosporin	Cefazolin	40 *R* (27.2)	*bla*_TEM-1_ (82.5), *bla*_CTX-M-55_ (15.0), *bla*_LAP-2_ (7.5), *bla*_CMY-2_ (5.0), *bla*_DHA-1_ (2.5)
	Cephalosporin	Cefuroxime	9 *R* (6.1)	*bla*_CTX-M-55_ (66.7), *bla*_TEM-1_ (55.6), *bla*_LAP-2_ (22.2), *bla*_CMY-2_ (22.2), *bla*_DHA-1_ (11.1)
	Cephalosporin	Ceftazidime/Cefotaxime^e^	8 *R* (5.4), 1 *I* (0.7)	*bla*_CTX-M-55_ (75.0), *bla*_CMY-2_ (25.0)
	Cephalosporin	Cefepime	6 *R* (4.1)	*bla*_CTX-M-55_ (100.0), *bla*_TEM-1_ (33.3), *bla*_CMY-2_ (33.3)
	Cephamycin	Cefoxitin	5 *R* (3.4)	*bla*_TEM-1_ (80.0), *bla*_CTX-M-55_ (40.0), *bla*_CMY-2_ (40.0), *bla*_LAP-2_ (20.0), *bla*_DHA-1_ (20.0)
	Fluoroquinolone	Ciprofloxacin	15 *R* (10.2), 54 *I* (36.7)	*gyrA* mutations (26.7); *qnrS1* (63.8), and others^f^
	Aminoglycoside	Amikacin	3 *R* (2.0)	*rmtB* (100)
	Carbapenem	Imipenem/Meropenem/Ertapenem	0 *R* (0)	Not detected
	Polymyxin	Colistin/Polymyxin B	147 *I* (100)^g^	*mcr-9.1* (0.7, silent)
	Tetracycline	Tigecycline	1 *R* (0.7)	–^h^
	β-lactam/BI	Ceftazidime/avibactam	0 *R* (0)	–
Summary	MDR (≥3 classes)		92 (62.6)	–

^a^Resistant (R) and intermediate (I) data are shown as number of isolates (percentage of total isolates). The prevalence of key genetic determinants is calculated using the corresponding number of resistant and/or intermediate isolates as the denominator, and is shown in parentheses.

^b^The two ceftiofur-intermediate isolates carried *bla*_DHA-1_ and *bla*_LAP-2_, respectively.

^c^Other genes detected in trimethoprim-sulfamethoxazole-resistant isolates include *dfrA27* (29.4%), *dfrA14* (7.4%), and *dfrA19* (1.5%).

^d^Other genes detected in chloramphenicol-resistant isolates include *catII* (9.1%), *catB3* (5.2%), *cmlA5* (2.6%).

^e^The same eight isolates were resistant to both ceftazidime and cefotaxime (5.4%), and the same one isolate was intermediate to both (0.7%).

^f^Other PMQR genes detected in ciprofloxacin-resistant/intermediate isolates include *qnrB6* (5.8%), *qnrB10* (4.3%), *qnrB4* (1.4%), *qnrS2* (1.4%).

^g^All 147 isolates were intermediate to polymyxins (no CLSI susceptible category); only one isolate carried a silent *mcr-9.1*.

^h^The tigecycline-resistant isolate carried no known plasmid-mediated resistance genes.

Tetracycline resistance was the most common, observed in 99 isolates (67.3%), followed by resistance to streptomycin (94 isolates, 63.9%), ampicillin (87 isolates, 59.2%), chloramphenicol (77 isolates, 52.4%), florfenicol (74 isolates, 50.3%), and trimethoprim-sulfamethoxazole (68 isolates, 46.3%). All isolates remained susceptible to carbapenems. For polymyxins, all 147 isolates showed intermediate susceptibility. One isolate (0.7%) was resistant to tigecycline.

#### 3.3.1 β-Lactam resistance

Ampicillin resistance correlated with widespread carriage of *bla*_TEM-1_ (73 isolates, 83.9%). Resistance to ceftazidime, cefotaxime, and ceftiofur was identified in the same eight isolates (5.4%), with six carrying *bla*_CTX-M-55_ and two carrying *bla*_CMY-2_. One isolate carrying *bla*_DHA-1_ showed intermediate susceptibility to these agents but resistance to cefoxitin, and another carrying *bla*_LAP-2_ alone showed intermediate susceptibility to ceftiofur. Cefoxitin resistance was observed in five isolates (3.4%): three carried *bla*_CMY-2_ or *bla*_DHA-1_, and two carried *bla*_CTX-M-55_ alone. Regarding β-lactam/β-lactamase inhibitor combinations, 61 of the 87 ampicillin-resistant isolates (70.1%) were also resistant to ampicillin/sulbactam, whereas all eight ceftazidime-resistant isolates were susceptible to ceftazidime/avibactam.

#### 3.3.2 Aminoglycoside resistance

Streptomycin resistance (94 isolates, 63.9%) was mediated by *aph(6)-Id, aadA, aadA2, aph(3*″*)-Ib*, and *aadA16*. Gentamicin resistance was observed in 32 isolates (21.8%), with *aac(6*′*)-Iaa*, aac(3)-IId, aph(6)-Id, and *aph(3*″*)-Ib* as the main determinants. The 16S rRNA methyltransferase gene *rmtB* was identified in three isolates (2.0%), which were the only isolates resistant to amikacin and exhibited high-level gentamicin resistance.

#### 3.3.3 Phenicol resistance

Resistance to chloramphenicol and florfenicol was predominantly driven by *floR*. Additional chloramphenicol resistance genes included *cmlA1, catII, catB3*, and *cmlA5*.

#### 3.3.4 Folate pathway inhibitor resistance

Among the 68 trimethoprim-sulfamethoxazole-resistant isolates, *sul2, sul3, sul1*, and *dfrA12* were the most prevalent resistance genes .

#### 3.3.5 Fluoroquinolone resistance

Ciprofloxacin resistance was detected in 15 isolates (10.2%), and intermediate susceptibility in 54 isolates (36.7%). Among the 15 resistant isolates, *gyrA* mutations were identified in four; among the 54 intermediately susceptible isolates, PMQR genes (primarily *qnrS1*) were present in 48. The multidrug efflux pump gene *mdtK* was present in all 147 isolates.

#### 3.3.6 Last-line resistance genes

No carbapenemase genes were detected, consistent with the universal susceptibility to carbapenems. All 147 isolates showed intermediate susceptibility to polymyxins. The mobile colistin resistance gene *mcr-9.1* was identified in one isolate (0.7%). One tigecycline-resistant isolate (0.7%) carried no known plasmid-mediated tigecycline resistance genes.

### 3.4 Genomic epidemiology: evidence for transmission of high-risk clones

**Figure 2:**
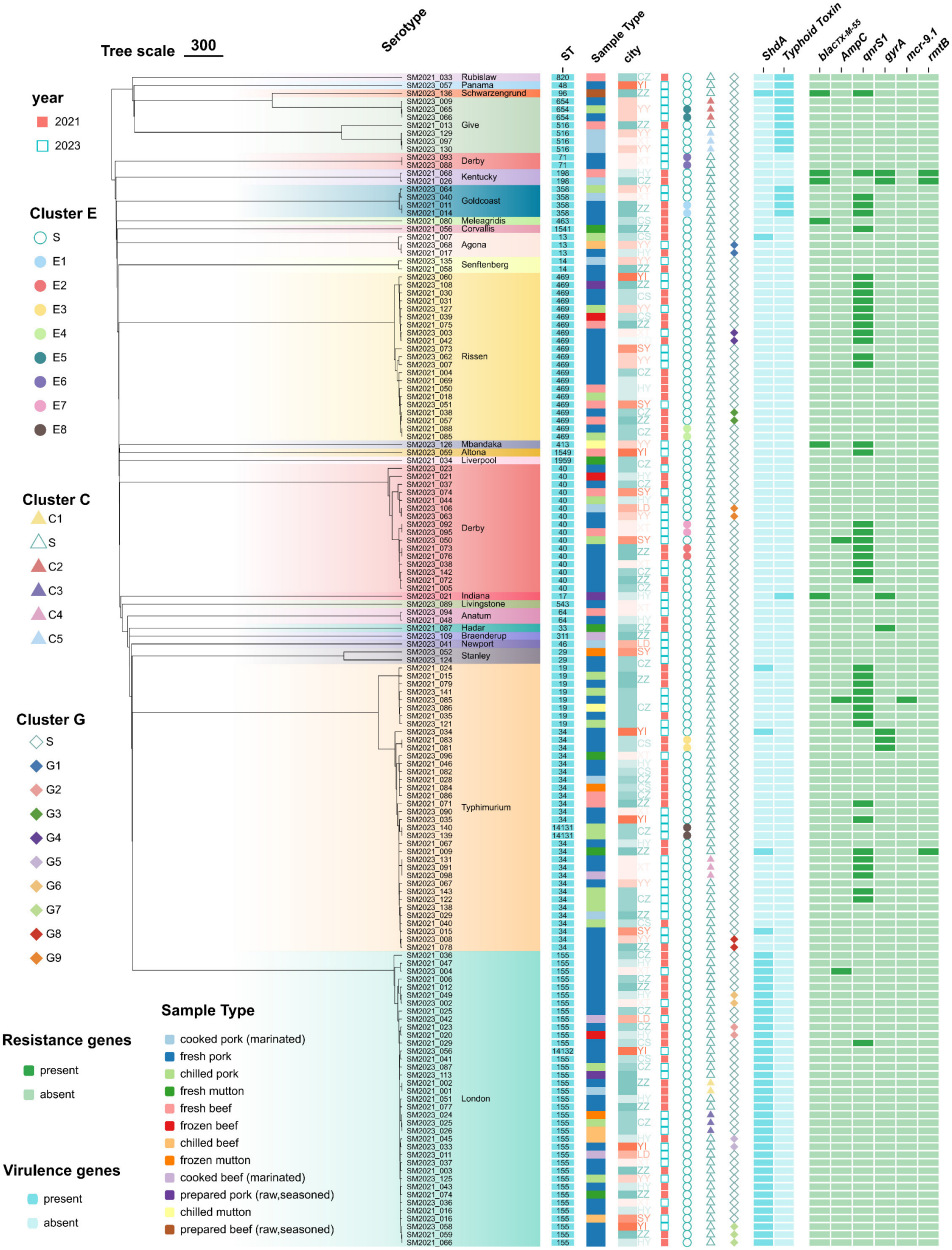
Phylogenetic relationships and distribution of key virulence and resistance genes among 147 *Salmonella* isolates. The annotations include: cgMLST cluster type (E: potential epidemiologically linked; C: common source-supported; G: genomic only; S: singleton/unclustered), serotype (color-coded, with text labels), ST type (numeric), sampling city (color-coded, abbreviated), sampling year (2021/2023), and sample type (color-coded). The heatmap shows the presence (dark) or absence (light) of selected virulence and resistance genes: typhoid toxin, *shdA, bla*_CTX-M-55_, AmpC (*bla*_CMY-2_/*bla*_DHA-1_), *rmtB, qnrS1, gyrA, and mcr-9.1*.

CgMLST analysis was performed to investigate the genetic relatedness among the 147 *Salmonella* isolates. Using a threshold of ≤ 10 allele differences to define genomic clusters, we identified 22 clusters, which were categorized into three types based on available sampling information: potential epidemiologically linked clusters (E), common source-supported clusters (C), and genomic clusters only (G) (Table 4; Figures 1, 2).

**Table 4:** Characteristics of genomic clusters identified by cgMLST analysis.

Cluster	Category	No.	Alleles	Epidemiological context	Interpretation
E1^*^	Linked	2	0	ZZ, same market; different vendors (same-day: 16 June 2021)	Market-level same-day point-source
E2^*^	Linked	2	0	ZZ, same market; different vendors (same-day: 20 October 2021)	Market-level same-day point-source
E3^*^	Linked	2	0	CS, same county (same-day: 25 October 2021)	County-level same-day exposure
E4^*^	Linked	2	1	CZ, same town (same-day: 2 November 2021)	Town-level same-day source
E5^*^	Linked	2	0–4	YY city (same-day: 6 June 2023); different counties	City-level same-day multi-county outbreak
E6^*^	Linked	2	0	XT city (same-day: 5 July 2023); different districts	City-level same-day exposure
E7^*^	Linked	2	1	XT, same district (same-day: 5 July 2023)	District-level same-day exposure
E8^*^	Linked	2	1	CZ, same street (same-day: 23 October 2023)	Street-level same-day local network
C1^*^	Common source	2	0	ZZ city (consecutive days, March 2021); two different areas	Localized temporal cluster
C2^*^	Common source	3	0–4	YY city (March and June 2023); three different areas	Persistent regional source
C3^*^	Common source	3	1–2	CZ city (consecutive days, March); three different areas; cold-chain	Localized temporal cluster
C4^*^	Common source	3	0–1	XT city (July and October 2023); two different areas; pork/beef	Persistent regional source
C5	Common source	3	1–5	XT (July) ↔ YY (September); different labs; cooked marinated meat	Regional common source
G1	Genomic	2	6	HY (2021) ↔ YY (2023); different labs	Long-term persistent lineage
G2	Genomic	2	9	HY (2021) ↔ CZ (2021); different labs	Widespread stable lineage
G3	Genomic	2	2	CZ (2021) ↔ ZZ (2021); different labs	Widespread stable lineage
G4	Genomic	2	8	CD (2021) ↔ XT (2023); different labs	Long-term persistent lineage
G5	Genomic	2	10	HY (2021) ↔ YI (2023); different labs	Regional background lineage
G6	Genomic	2	6	HY (2021) ↔ XT (2023); different labs	Regional background lineage
G7	Genomic	3	0–2	ZZ (2021) ↔ HY (2021) ↔ YI (2023); different labs	Widespread stable lineage
G8	Genomic	2	8	ZZ (2021) ↔ YY (2023); different labs	Long-term persistent lineage
G9	Genomic	2	5	YY (June) ↔ LD (August); different labs	Widespread stable lineage

Interpretive caution: For clusters marked with^*^, isolates were processed by the same laboratory.

XT, Xiangtan; YY, Yueyang; CZ, Chenzhou; ZZ, Zhuzhou; CS, Changsha; LD, Loudi; CD, Changde; HY, Hengyang; YI, Yiyang; ↔, connection between locations.

#### 3.4.1 Potential epidemiologically linked clusters (E)

Eight clusters were identified in this category, comprising isolates with clear spatiotemporal connections (e.g., same market or same-day sampling). Cluster E1 and E2 were recovered from the same market but different vendors. Cluster E4 and E8 were recovered from isolates collected within the same town or street. The remaining E clusters (E3, E5, E6, E7) comprised isolates collected on the same day from different districts or counties of the same city.

#### 3.4.2 Common source-supported clusters (C)

Five clusters were assigned to this category, comprising isolates with high genomic similarity recovered from different time points or different sampling sites, but lacking strong spatiotemporal links (e.g., same-day sampling or same-market exposure). Cluster C5 linked three isolates from Xiangtan (July 2023) and Yueyang (September 2023) processed by different laboratories (1–5 allele differences), all recovered from cooked marinated meat products. Cluster C3 comprised three cold-chain samples from three different areas of Chenzhou collected on consecutive days (frozen mutton, chilled pork, chilled beef) with 1–2 allele differences. Cluster C2 contained three isolates from three different areas of Yueyang City collected in March and June 2023 with 0–4 allele differences. Cluster C1 comprised two isolates from two different areas of Zhuzhou City collected on consecutive days in March 2021. Cluster C4 contained three isolates recovered from pork and beef products collected from two different areas of Xiangtan City in July and October 2023.

#### 3.4.3 Genomic clusters only (G)

Nine clusters were assigned to this category, comprising isolates with genomic similarity ( ≤ 10 allele differences) but no discernible epidemiological links. Notably, Cluster G7 linked isolates from Zhuzhou (2021), Hengyang (2021), and Yiyang (2023) with 0–2 allele differences. Cluster G2 (Hengyang 2021 ↔ Chenzhou 2021) and Cluster G4 (Changde 2021 ↔ Xiangtan 2023) also linked isolates from different cities and years.

#### 3.4.4 Convergence of high-risk traits in specific clusters

Analysis of the distribution of key resistance and virulence genes across the 22 genomic clusters revealed distinct patterns of association (Table 4; Figure 2). The typhoid toxin gene cluster (*cdtB, pltA, pltB*) was detected in multiple clusters across different transmission categories, including potential epidemiologically linked clusters E1 (*S*. Goldcoast) and E5 (*S*. Give), the common source-supported cluster C2 (*S*. Give), and the regional cluster C5 (*S*. Give). In contrast, the intestinal persistence factor *shdA* showed a strong lineage-specific association, being predominantly identified with the dominant *S*. London ST155 clone across genomic background clusters (G2, G5, G6, G7) and common source-supported clusters (C1, C3). The plasmid-mediated quinolone resistance (PMQR) gene *qnrS1* was widely distributed across clusters E1, E2, E7, G4, and C4. Other clinically significant resistance genes, including the ESBL gene *bla*_CTX-M-55_, the AmpC genes *bla*_CMY-2_ and *bla*_DHA-1_, and the 16S rRNA methyltransferase gene *rmtB*, were not consistently associated with specific genomic clusters.

## 4 Discussion

### 4.1 Virulence gene profiles and pathogenic implications

NTS remains a leading cause of foodborne bacterial diseases worldwide, with infections ranging from self-limiting gastroenteritis to life-threatening invasive disease, particularly in children, the elderly, and immunocompromised individuals (Havelaar et al., 2015). In this study, we systematically characterized the virulence gene repertoire of 147 NTS isolates from retail red meat, representing 24 serotypes and 29 sequence types.

#### 4.1.1 Lineage-specific association of shdA with S. London

The exclusive presence of *shdA* in all 37 *S*. London isolates (36 ST155 and 1 ST14132) is a striking finding. *ShdA* is an autotransporter protein that mediates binding to fibronectin, promoting prolonged intestinal colonization and fecal shedding in mice (Kingsley et al., 2000, 2002). However, its role may be host-specific, as studies in pigs suggest limited contribution to persistence (Boyen et al., 2006). This apparent paradox does not diminish the significance of *shdA* as a stable genetic marker of the dominant *S*. London lineage. From a One Health perspective, the high prevalence of *shdA* in *S*. London isolates underscores the lineage's capacity for widespread circulation in the food supply chain, regardless of the precise molecular mechanism in any single host.

#### 4.1.2 Emergence of typhoid toxin genes in NTS

Of major concern, the complete typhoid toxin gene cluster (*cdtB, pltA, pltB*) was identified in 14 NTS isolates (9.5%) across diverse serotypes, including *S*. Give, *S*. Goldcoast, *S*. Rubislaw, *S*. Schwarzengrund, and *S*. Indiana. Typhoid toxin, a potent A_2_*B*_5_ toxin with a unique structure (one CdtB subunit, one PltA subunit, and five PltB subunits), has long been considered exclusive to typhoidal serovars (*S*. Typhi and *S*. Paratyphi *A*) and plays a critical role in the pathogenesis of typhoid fever (Galán, 2016). Its detection in foodborne NTS isolates therefore provides compelling evidence for horizontal gene transfer from typhoidal to non-typhoidal serovars, challenging the traditional paradigm that confines this virulence module to typhoidal lineages (Miller et al., 2018; Delgado-Suárez et al., 2018). NTS strains harboring the typhoid toxin gene cluster could potentially cause more severe disease than typical NTS infections, particularly in vulnerable populations. Experimental studies have demonstrated that typhoid toxin produced by certain NTS serotypes induces DNA damage and contributes to systemic spread in animal models (Miller et al., 2018). From a public health perspective, this finding challenges conventional serotyping-based risk assessment and underscores the need for transitioning to genomic surveillance, as virulence potential is increasingly recognized to be strain-specific rather than serovar-specific (Priya et al., 2023; Mohamed and Habib, 2025). If these toxin-encoding strains establish themselves in the food supply, they could pose an elevated public health risk, potentially causing outbreaks with higher rates of invasive disease—a particular concern given that immunocompromised individuals, children, and the elderly are at the highest risk for invasive non-typhoidal *Salmonella* infections (Piccini and Montomoli, 2020; Crump et al., 2015). The detection of a typhoidal-associated virulence module in foodborne NTS isolates thus reinforces the need for integrated surveillance across human, animal, food, and environmental compartments.

The defective cluster (containing only *cdtB* and *pltA*, but lacking *pltB*) in a single *S*. Panama isolate provides a valuable evolutionary insight. Since all three subunits are required for full toxin activity (Galán, 2016), this partial cluster likely encodes a non-functional product. This observation aligns with the inherent flexibility of AB_5_ toxin architecture, where the pentameric B subunit can be replaced or diversified through horizontal gene transfer (Fowler et al., 2019). Indeed, *S*. Typhi encodes an alternative B subunit, PltC, which assembles with the same A subunits to produce a second form of typhoid toxin, and *S*. Bongori independently evolved a distinct B subunit, PltD (Chemello and Fowler, 2024). These findings illustrate how the plasticity of AB_5_ toxins has fueled their evolutionary diversification and expansion (Fowler et al., 2019). Therefore, this partial cluster may represent an evolutionary intermediate—either incomplete acquisition captured during horizontal transfer, or evidence of ongoing gene loss—serving as a reservoir for future recombination events.

### 4.2 Antimicrobial resistance mechanisms and clinical implications

The high prevalence of antimicrobial resistance (78.2%) and multidrug resistance (62.6%) among *Salmonella* isolates from retail red meat poses significant public health challenges. While resistance to older antibiotics was widespread, the detection of resistance to critically important antimicrobials—particularly third-generation cephalosporins, fluoroquinolones, and last-line agents such as amikacin—is of major concern.

Resistance to third-generation cephalosporins was primarily mediated by the ESBL gene *bla*_CTX-M-55_ and the plasmid-mediated AmpC gene *bla*_CMY-2_. *bla*_CTX-M-55_ is a common ESBL variant in Chinese food-producing animals and has been increasingly reported in human isolates, highlighting zoonotic transmission through the food chain (Xiao et al., 2024; Wei et al., 2023). The co-occurrence of ceftiofur resistance with third-generation cephalosporin resistance underscores that veterinary cephalosporin use directly co-selects for resistance to human critically important cephalosporins. Regarding β-lactam/β-lactamase inhibitor combinations, a substantial proportion of ampicillin-resistant isolates remained resistant to ampicillin/sulbactam, whereas all ceftazidime-resistant isolates were susceptible to ceftazidime/avibactam, suggesting that the latter may represent a valuable carbapenem-sparing option for infections caused by ESBL- and AmpC-producing *Salmonella* (Isler et al., 2020).

Critically, the 16S rRNA methyltransferase gene *rmtB* was identified in three isolates (2.0%)—the only isolates resistant to amikacin—conferring pan-aminoglycoside resistance and compromising last-line treatment options for invasive salmonellosis (Doi and Arakawa, 2007; Obranić et al., 2024).

A gradient from intermediate susceptibility to full ciprofloxacin resistance was observed. PMQR genes (predominantly *qnrS1*) were highly prevalent among intermediately susceptible isolates (88.9%, 48/54), whereas *gyrA* mutations were strongly associated with full resistance (26.7%, 4/15). This distribution supports a stepwise resistance evolution model: PMQR genes confer low-level resistance, facilitating the subsequent selection of chromosomal mutations such as those in *gyrA*, which then lead to high-level resistance. However, direct acquisition of *gyrA* mutations can also occur independently, representing an alternative pathway (Robicsek et al., 2006; Jacoby et al., 2014).

No carbapenemase genes were detected, consistent with universal carbapenem susceptibility. The mobile colistin resistance gene *mcr-9.1* was identified in one isolate (0.7%) that remained phenotypically intermediate, consistent with reports that MCR-9 expression is often silent (Carroll et al., 2019). All isolates had polymyxin MIC values ≤ 2 μg/mL. According to CLSI breakpoints revised in 2020, a “susceptible” category was not defined for polymyxins due to PK/PD and toxicity concerns (Satlin et al., 2020); thus, these isolates are categorized as intermediate. This consistent intermediate phenotype likely reflects the intrinsic ability of *Salmonella* to modify lipid A via PmrAB/PhoPQ (Gunn et al., 1998; Olaitan et al., 2014). One tigecycline-resistant isolate (0.7%) carried no known plasmid-mediated resistance genes; its resistance may involve AcrAB-TolC overexpression or regulatory mutations (Hentschke et al., 2010; Li et al., 2024), warranting further investigation.

Widespread resistance to tetracycline, streptomycin, and sulfonamides reflects historical antibiotic use. The predominance of *sul2* and the co-carriage of *sul* and *dfrA* genes in most trimethoprim-sulfamethoxazole-resistant isolates highlight the role of mobile genetic elements in co-disseminating these determinants (Rådström and Swedberg, 1988; Blahna et al., 2006). Phenicol resistance was primarily driven by *floR*.

### 4.3 Genomic epidemiology and transmission patterns

#### 4.3.1 Transmission patterns

CgMLST analysis revealed a hierarchical structure of *Salmonella* dissemination in the retail meat supply chain, ranging from regional common sources to local point-source outbreaks. This multi-level transmission pattern aligns with the complex dynamics of foodborne pathogen circulation documented in recent genomic surveillance studies (Kuronen et al., 2025; Wang et al., 2025).

The identification of potential epidemiologically linked clusters (E) provided evidence for local transmission at the retail level, with isolates showing clear spatiotemporal proximity such as same market or sampling from the same city. These findings suggest that point-of-sale contamination—potentially mediated by shared equipment or handling practices—may contribute to the spread of *Salmonella* in retail settings. However, we acknowledge that same-day sampling could also reflect batch effects, and this interpretation should be considered cautiously (as noted in the Limitations).

Common source-supported clusters (C) comprised isolates with high genomic similarity but lacking strong spatiotemporal links. The presence of such clusters, particularly those spanning different cities and months, points to contamination from shared upstream sources (e.g., common distributors or processors) rather than local retail-level events. Notably, isolates from cold-chain products and cooked marinated meat products formed such clusters, indicating that dissemination through centralized distribution networks and refrigerated supply chains may play a critical role.

Genomic background lineages (G) consisted of isolates with genomic similarity but no discernible epidemiological links. The detection of clones persisting over several years and across multiple cities suggests that once established, certain lineages become endemic in regional reservoirs and serve as persistent sources of contamination.

#### 4.3.2 Convergence of high-risk traits in specific clusters

The distribution of key resistance and virulence genes across genomic clusters revealed distinct patterns. The typhoid toxin gene cluster was detected across multiple transmission categories (including both potential epidemiologically linked and common source-supported clusters), providing evidence for horizontal transfer of this virulence module among diverse non-typhoidal *Salmonella* lineages. This finding challenges the traditional paradigm that confines typhoid toxin to typhoidal serovars and raises concerns about the potential emergence of NTS strains with enhanced pathogenic potential (Delgado-Suárez et al., 2018; Priya et al., 2023). In contrast, the intestinal persistence factor *shdA* showed a strong lineage-specific association with the dominant *S*. London lineage, consistent with its role in promoting prolonged host colonization and vertical inheritance (Kingsley et al., 2002; Boyen et al., 2006). The plasmid-mediated quinolone resistance gene *qnrS1* was widely distributed across multiple clusters, reflecting its location on mobile genetic elements. Notably, one cluster co-carried both the typhoid toxin gene cluster and *qnrS1*, underscoring the potential for simultaneous dissemination of resistance and virulence determinants through the food chain. Other clinically significant resistance genes (e.g., *bla*_CTX-M-55_, *bla*_CMY-2_, *rmtB*) were not consistently associated with specific genomic clusters, suggesting that their dissemination is likely driven by plasmid-mediated horizontal gene transfer rather than clonal expansion.

#### 4.3.3 Public health implications

These findings have direct implications for food safety policy. The multi-level transmission patterns observed-from regional common sources to local outbreaks-highlight the need for integrated surveillance systems that combine genomic and epidemiological data across the entire food production continuum. Targeted interventions at centralized processing and distribution nodes, coupled with improved hygiene practices at retail points, are essential to interrupt transmission. The convergence of high-risk resistance and virulence determinants in disseminating lineages underscores the urgency of implementing such measures to prevent the establishment and spread of clinically significant clones in the food supply.

### 4.4 Limitations and future perspectives

This study has several limitations. First, bacterial isolation was performed by multiple municipal laboratories. Although all sampling and laboratory procedures were performed by trained personnel following standardized protocols, the risk of cross-contamination during sample collection or laboratory processing cannot be completely excluded in a multi-center surveillance system, particularly for clusters with same-day or same-market sampling processed by the same laboratory (these clusters are indicated in the table footnotes). Second, the classification of genomic clusters into E (potential epidemiologically linked), C (common source-supported), and G (genomic clusters only) was based on available spatiotemporal information. The use of “same-day sampling” as an indicator of potential epidemiological linkage should be interpreted with caution, as it may reflect sampling or laboratory processing batch effects rather than true transmission. Third, samples were collected only at the retail level, precluding source tracing to upstream stages such as farming, slaughtering, and distribution, which limits precise source attribution. The detection of cold-chain associated clusters (C3) and persistent lineages (C2, C4) suggests that contamination may originate earlier in the production chain, but confirmation requires integrated sampling across the entire farm-to-fork continuum. Fourth, reliance on short-read sequencing may limit the resolution of certain mobile genetic elements, particularly plasmids carrying antimicrobial resistance genes. Long-read sequencing approaches would enable complete assembly of plasmid structures and more accurate tracking of horizontal gene transfer events. Fifth, silent resistance genes (e.g., *mcr-9.1*) highlight the need for complementary approaches (e.g., transcriptomics, proteomics) to fully elucidate resistance mechanisms.

This study represents the first part of a comprehensive “One Health” investigation of *Salmonella* in Hunan Province, focusing on isolates from retail red meat. A large collection of clinical *Salmonella* isolates from the same geographic region and overlapping time period, including those from confirmed outbreak strains, has already been assembled. Comparative genomic analysis between these foodborne and clinical isolates is currently underway and will enable us to: (1) determine the extent to which the high-risk clones identified in retail meat contribute to human infections; (2) trace transmission pathways from farm to clinic; and (3) identify clone-specific risk factors for human disease. The results of this ongoing work will be reported in a forthcoming manuscript.

## 5 Conclusions

This study of 147 *Salmonella* isolates from retail red meat in Hunan Province revealed high multidrug resistance (62.6%) and clinically significant resistance genes, including *rmtB, bla*_CTX-M-55_, and *bla*_CMY-2_. The complete typhoid toxin gene cluster was identified in 14 NTS isolates (9.5%) across multiple serotypes, challenging the traditional paradigm that restricts this toxin to typhoidal serovars. CgMLST analysis revealed multi-level transmission patterns, from regional common sources to local outbreaks. These findings underscore the urgent need for integrated One Health surveillance of antimicrobial resistance and virulence determinants across the food chain. Ongoing comparative genomic analysis with clinical isolates will further assess the contribution of foodborne high-risk clones to human disease.

## Data Availability

The datasets presented in this study can be found in online repositories. The names of the repository/repositories and accession number(s) can be found at: https://ngdc.cncb.ac.cn/gsa/.
